# Lumpectomy with or without postoperative radiotherapy for breast cancer with favourable prognostic features: results of a randomized study

**DOI:** 10.1054/bjoc.2000.1571

**Published:** 2001-01

**Authors:** K Holli, R Saaristo, J Isola, H Joensuu, M Hakama

**Affiliations:** Department of Palliative Medicine, University Hospital and University of Tampere; Department of Surgery, University Hospital of Tampere; Department of Pathology, University Hospital of Tampere; Department of Oncology, University Hospital of Helsinki; The School of Public Health, University of Tampere, Finland

**Keywords:** low risk breast cancer, conservative surgery, radiotherapy

## Abstract

The aim of this trial was to study the value of adding post-operative radiotherapy to lumpectomy in a subgroup of breast cancer patients with favourable patient-, tumour-, and treatment-related prognostic features. 152 women aged over 40 with unifocal breast cancer seen in preoperative mammography were randomly assigned to lumpectomy alone (no-XRT group) or to lumpectomy followed by radiotherapy to the ipsilateral breast (50 Gy given within 5 weeks, XRT group). All cancers were required to be invasive node-negative, smaller than 2 cm in diameter and well or moderately differentiated, to contain no extensive intraductal component, to be progesterone receptor-positive, DNA diploid, have S-phase fraction ≤7 and be excised with at least 1 cm margin. During a mean follow-up time of 6.7 years, 13 (18.1%) cancers recurred locally in the no-XRT and 6 (7.5%) in the XRT group (*P* = 0.03). There was no difference between the groups in the ultimate breast preservation rate (95.0% vs. 94.4% in XRT and no-XRT, respectively, *P* = 0.88), distant metastasis-free survival (*P* = 0.36), or 5-year cancer-specific survival (97.1% in XRT and 98.6 in no-XRT). Radiation therapy given after lumpectomy reduces the frequency of ipsilateral breast recurrences even in women with small breast cancer with several favourable clinical and biological features. However, the breast preservation rate may not increase due to more frequent use of salvage mastectomies in patients treated with postoperative radiotherapy. © 2001 Cancer Research Campaign http://www.bjcancer.com


*British Journal of Cancer* (2001) **84**(2), 164–169
© 2001 Cancer Research Campaign
doi: 10.1054/ bjoc.2000.1571, available online at http://www.idealibrary.com on IDEAL®

### Lumpectomy with or without postoperative radiotherapy for breast cancer with favourable prognostic features: results of a randomized study

**K Holli[1], R Saaristo[2], J Isola[3], H Joensuu[4] and M Hakama[5]**

[1]Department of Palliative Medicine, University Hospital and University of Tampere; [2]Department of Surgery, University Hospital of Tampere; [3]Department of Pathology, University Hospital of Tampere; [4]Department of Oncology, University Hospital of Helsinki; [5]The School of Public Health, University of Tampere, Finland

**Summary** The aim of this trial was to study the value of adding post-operative radiotherapy to lumpectomy in a subgroup of breast cancer patients with favourable patient-, tumour-, and treatment-related prognostic features. 152 women aged over 40 with unifocal breast cancer seen in preoperative mammography were randomly assigned to lumpectomy alone (no-XRT group) or to lumpectomy followed by radiotherapy to the ipsilateral breast (50 Gy given within 5 weeks, XRT group). All cancers were required to be invasive node-negative, smaller than 2 cm in diameter and well or moderately differentiated, to contain no extensive intraductal component, to be progesterone receptor-positive, DNA diploid, have S-phase fraction ≤7 and be excised with at least 1 cm margin. During a mean follow-up time of 6.7 years, 13 (18.1%) cancers recurred locally in the no-XRT and 6 (7.5%) in the XRT group ($P = 0.03$). There was no difference between the groups in the ultimate breast preservation rate (95.0% vs. 94.4% in XRT and no-XRT, respectively, $P = 0.88$), distant metastasis-free survival ($P = 0.36$), or 5-year cancer-specific survival (97.1% in XRT and 98.6 in no-XRT). Radiation therapy given after lumpectomy reduces the frequency of ipsilateral breast recurrences even in women with small breast cancer with several favourable clinical and biological features. However, the breast preservation rate may not increase due to more frequent use of salvage mastectomies in patients treated with postoperative radiotherapy. © 2001 Cancer Research Campaign http://www.bjcancer.com

**Keywords**: low risk breast cancer; conservative surgery; radiotherapy

Several randomized studies (Clark et al, 1992; Veronesi et al, 1993; Liljegren et al, 1994; Fisher et al, 1995; Veronesi et al, 1995; Forrest et al, 1996; Liljegren et al, 1996; Renton et al 1996) and a meta-analysis (Early Breast Cancer Trialists Collaborative Group, 1995) have concluded that lumpectomy or quadrantectomy result in survival rates similar to those in mastectomy in stage I and II breast cancer, provided that adequate postoperative breast irradiation using modern radiotherapy techniques is given subsequent to breast preserving surgery. The main objective for postoperative radiotherapy is to reduce the rate of locoregional recurrences. The rate of ipsilateral breast recurrences has been reported to vary between 9% and 35% in patients not receiving postoperative irradiation after breast-preserving surgery. Postoperative radiation reduces the local recurrence rate to about one third (Kurtz et al, 1989; Clark et al, 1992; van Dongen et al, 1992, Veronesi et al, 1993; Liljegren et al, 1994; Early Breast Cancer Trialists Collaborative Group, 1995; Fisher et al, 1995; Veronesi et al, 1995; Forrest et al, 1996; Liljegren et al, 1996; Renton et al 1996), and it has reduced the risk of recurrence after breast-preserving surgery in randomized studies regardless of whether tumours 2 cm or smaller (Liljegren et al, 1999), tumours smaller than 2.5 cm (Veronesi et al, 1993), or 4 cm or smaller in diameter (Clark et al, 1992; Fisher et al, 1995; Forrest et al, 1996) had been accrued, and regardless of whether only invasive or purely intraductal cancers were included in the studies (Fisher et al, 1998).

There are at present no established clinical or biological factors that reliably identify patients who could be spared from postoperative radiotherapy after lumpectomy (Fourquet et al, 1989; Locker et al, 1989; Clarke and Martinez, 1992; Julien et al, 2000). The most important risk factors for local recurrence after breast-preserving surgery are the width of the surgical margin and the presence of an extensive intraductal component (EIC) (Kurtz, 1992; Gage et al, 1996; Sinn et al, 1998; Voogd et al, 1999) Gross resection margins of 1 to 2 cm are generally considered necessary (Gage et al, 1996; Renton et al, 1996), but in one study approximately 30% of patients with EIC-positive cancers had prominent residual intraductal carcinoma at least 2 cm beyond the edge of the primary tumour, compared to only 2% of EIC-negative cancers (Holland et al, 1990), suggesting that even a 2-cm margin may be inadequate unless radiotherapy is given. The lowest local relapse rates were seen in the QUART study where there was the most extensive surgery (Veronesi et al, 1985). Although evidence regarding other risk factors is more controversial, it has been suggested that young age at presentation (Clark et al, 1992; Kurtz, 1992; de la Rochefordiere et al, 1993; Fowble et al, 1994), high nuclear grade, high tumour proliferation rate, presence of vascular invasion (Kurtz et al, 1990; Clark et al, 1992; Dalberg et al, 1999) and multifocality predict local recurrence.

Breast irradiation also has adverse effects such as an increased risk of breast fibrosis and oedema, nipple retraction, teleangiectasia and other skin changes, and impaired cosmesis, in particular if large daily fractions or if total target doses higher than 50 Gy are

*Received 15 June 2000*
*Revised 7 September 2000*
*Accepted 11 October 2000*

*Correspondence to: K Holli*

given to the entire breast volume (de al Rochefordiere et al, 1992). Postoperative radiotherapy also increases the economic cost of treatment. If therefore recurrence rates were similar between radiotherapy and no-radiotherapy groups, radiation should be avoided. We hypothetized that if breast-conserving surgery is based on standardized surgical procedures and carried out by a small team of trained breast cancer surgeons, and if only small and biologically non-aggressive cancers are selected for lumpectomy, the rate of breast cancer recurrence might remain low even without postoperative radiotherapy. Here we report the results of a randomized trial in this selected breast cancer subgroup, where the patients were randomly assigned either to postoperative radiotherapy (XRT) or to no radiotherapy (no-XRT) after mammography and lumpectomy. The estimation of tumour-biologic aggressiveness was based on a combination of several prognostic features often considered to be favourable. To our knowledge, in all randomized studies reported so far patient selection for breast-preserving therapy has been primarily based on tumour size and not on factors related to tumour-biological aggressiveness, which might be a relevant approach in view of the tumour tendency to recur locally.

### MATERIALS AND METHODS

### Patients

All conservatively operated female patients with invasive breast cancer treated in the Tampere University Hospital District between May 1990 and May 1995 were counseled by an oncologist 3 to 6 weeks after surgery. After counseling and informed consent, patients who were considered to have breast cancer with a low risk of recurrence were randomly assigned either to receive or not to receive postoperative radiotherapy. All patients had a mammogram taken preoperatively. Low risk of recurrence was strictly defined, and for study accrual all patient-related, tumour-related and treatment-related factors were to be fulfilled. Age at randomization was to be over 40, and patients with axillary nodal or distant metastases at presentation were excluded. The greatest diameter of the tumour measured microscopically was required to be smaller than 20 mm, histological grade either I (well differentiated) or II (moderately differentiated), progesterone receptor status positive, no extensive intraductal component (EIC) present, nuclear DNA content diploid as determined by DNA flow cytometry, the S-phase fraction low (≤7%), and the tumour unifocal in preoperative mammography. The resection margins were required to be free of cancer with at least 1 cm healthy breast tissue between cancer and resection margin under light microscopy. The type of surgery to be carried out was lumpectomy with axillary dissection. If the tumour size was too small to allow sampling for hormone receptor and flow cytometric DNA content assays (usually less than 5 mm, 10% of cases), histologic grade I or II was considered sufficient evidence for low biological aggressiveness (Table 1).

### Breast surgery

Segmental resection and axillary dissection were carried out according to standardized procedures. 93% of all breast cancer surgery undertaken was performed by 4 surgeons. The mammary gland was dissected free in the plane of Scapa’s fascia down to the pectoral muscle. The pectoral fascia was included in the removed specimen. Lumpectomy was effected with bi-digital control of the palpable or localized tumours. Non-palpable tumours were localized with radiological wire-hook marking. The resection specimen was removed with at least 1 cm gross tumour-free margins measured by pathologist. A level I and level II lymph node dissection was performed through a separate axillary incision.

### Randomization

Randomization was applied 3 to 8 weeks after surgery. After obtaining informed consent, assignment to the treatment groups was carried out by a phone call to the secretary, located at the Department of Oncology. The participant’s name and social security number, comprising date of birth and an individual identification code, was provided for unequivocal identification. Randomization between the 2 arms was carried out by a computer program generating random digits. No stratification was done.

### Postoperative radiotherapy and concomitant therapy

In the group assigned to postoperative radiotherapy, this was started within a few days after randomization and within 4 to 8 weeks after surgery. Radiotherapy was given by linear accelerator using 2 opposed tangential fields with 5 MeV photons. The total radiation dose was 50 Gy given within 5 weeks and in 2-Gy daily fractions. The dose was specified at the International Commission on Radiation Units and Measurements (IRCU) reference point located at the intersection point of the 2 opposing beams within the planning target volume, which comprised the entire ipsilateral breast volume and the underlying chest wall but not the regional axillary lymph nodes. Dose inhomogeneity within the target volume was below 10%. No booster dose was given to the tumour site.

None of the patients received adjuvant endocrine therapy or chemotherapy. Any medication considered necessary for treatment of diseases other than breast cancer was allowed.

### Ethical considerations

The study protocol was accepted by the Ethical Committee of the hospital, and written informed consent was required before accrual.

### Follow-up

All patients were regularly followed up at the out-patient department of the hospital. None was lost from follow-up. The follow-up visits took place every 3 months during the first year after randomization, every 4 months during the second year, and every 6 months thereafter. At study closure in April 30th 1999, the mean follow-up time for patients still alive was 6.7 years (range, 3.8 to 9.2 years). Clinical examination was made of the breasts and blood chemistry analysed at every follow-up visit; mammography was performed at 1-to-2-year intervals.

### Statistical analysis

Survival was calculated from the date of surgery to death or the last day of follow-up for those patients still alive. Time to locoregional progression was defined as the time from operation until cancer was found in the ipsilateral breast or the regional lymph nodes, and time to distant progression as the time from

© 2001 Cancer Research Campaign randomization to the appearance of metastases outside the loco-regional area. Cancer-specific survival was obtained by censoring deaths from intercurrent causes. Survival times were examined by the Kaplan-Meier product-limit method, and survival between the treatment arms was compared using log-rank test. Frequency tables were analysed using the chi-square test with continuity correction factor or Fisher's exact test. All *P* values were 2-tailed.

### RESULTS

A total of 152 patients were enrolled for the study, of whom 80 were assigned to receive post-lumpectomy radiotherapy and 72 to no further therapy. One patient randomized to receive radiotherapy refused treatment and was excluded. The two arms were well balanced, and there were no differences between the treatment groups in respect of age at diagnosis, cancer histological type or grade, tumour size or S-phase fraction size (Table 2). There was no association of age at diagnosis ($P = 0.58$) or tumour size ($P = 0.95$), histologic type ($P = 0.94$) or histologic grade ($P = 0.96$) with the local recurrence rate in the series (Table 3).

19 (12.5%) ipsilateral recurrences were diagnosed during follow-up. 13 (18.1%) of these were found in the no-radiotherapy group, and 6 (7.5%) in the radiotherapy group. The 5-year loco-regional disease-free survival was 93.7% when postoperative radiotherapy was given and 85.9% when not ($P = 0.03$) (Figure 1). All locoregional recurrences were located in the preserved ipsilateral breast. After recurrence, mastectomy was carried out in 4 (31%) of the 13 cases of locoregional recurrence in the no-radio-therapy group and a re-resection was done in the remaining 9. In the radiotherapy group 4 (67%) of the 6 recurrences were treated with mastectomy and only 2 with re-resection ($P = 0.32$). Radiotherapy was given after re-resection to patients randomized to the no-radiotherapy group. Hence, the breast preservation rate was almost identical in the two groups, 76 (95.0%) out of 80 in the radiotherapy group and 68 (94.4%) out of 72 in the no-radio-therapy group, ($P = 0.88$).

Distant metastases were found in only 9 (5.9%) patients, in 7 (8,8%) patients who received postoperative radiotherapy and in 2 (2.8%) of those who did not ($P = 0.41$) (Fig. 2). When recurrence-free survival (RFS) was computed taking into account both loco-regional and distant recurrences, no significant difference in recurrence-free survival was found between the groups. The 5-year RFS was 86.1% for patients in the radiotherapy group and

**Table 1** Inclusion criteria for the study

| Category | Parameter |
|---|---|
| Patient-related | Age at randomization >40 |
| Tumour-related | No axillary nodal metastases (pN0) |
| | No distant metastases (M0) |
| | Primary tumour size <2 cm |
| | No extensive intraductal component (EIC-) |
| | No multicentricity or multifocality in preoperative mammogram |
| | Histological grade I or II |
| | Progesterone receptor (PgR) positive |
| | If tumour size <5 mm, histological grade I or II |
| Treatment-related | Lumpectomy and axillary dissection performed |
| | Microscopical tumour-free margin 1 cm or greater |

**Table 2** Patient and tumour characteristics

| Parameter | Radiation therapy (*n* = 80) | Controls (–) (*n* = 72) |
|---|---|---|
| Median age at diagnosis (range) | 54 (42–73) | 56 (40–76) |
| Primary tumour size (mm) | | |
| ≤ 5 | 9 | 9 |
| 6–10 | 21 | 22 |
| 10–15 | 29 | 32 |
| 16–20 | 13 | 17 |
| Histological type (*n*) | | |
| ductal | 65 | 51 |
| lobular | 6 | 10 |
| special | 9 | 11 |
| Histological grade (n) | | |
| grade I | 52 | 52 |
| grade II | 10 | 19 |
| not specified | 10 | 9 |
| Median S-phase fraction (%) | 4.0 (1.0–7.9) | 4.0 (1.0–7.9) |

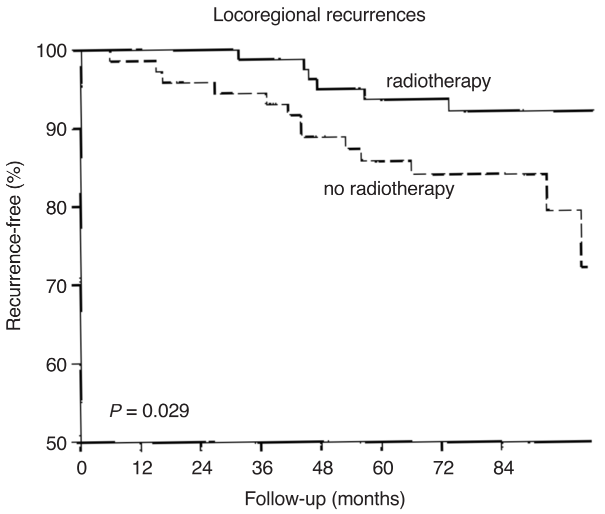

**Figure 1** Locoregional disease-free survival among 152 patients with low-risk breast cancer randomized either to postoperative radiotherapy (solid line, *n* = 80) or to no further therapy (dashed line, *n* = 72) after lumpectomy

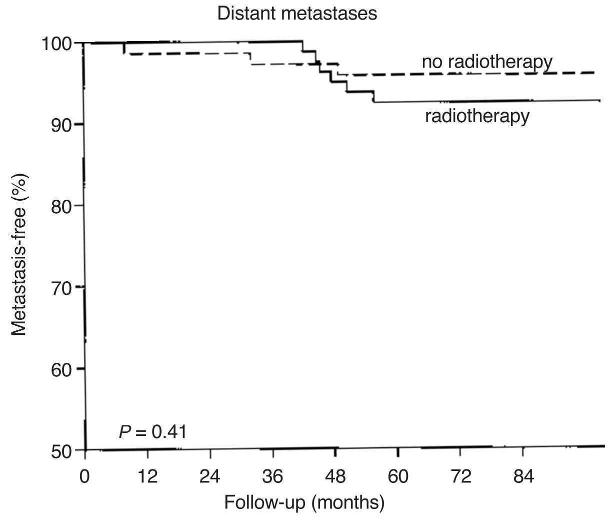

**Figure 2** Distant disease-free survival. Solid line, postoperative radiotherapy given; dashed line, no postoperative radiotherapy

 © 2001 Cancer Research Campaign

84.4% for those not receiving postoperative radiotherapy, (*P* = 0.36) (Fig. 3). Only 4 (2.6%) patients died of breast cancer during follow-up (2 in either group), thus resulting in a 5-year cancer-specific survival rate as high as 97.1% in the XRT group and 98.6% in no-XRT (Fig. 4).

### DISCUSSION

In this single-centre trial based on a geographically well-defined population, emphasis was placed on minimizing patient-related, tumour-related and treatment-related risk factors for breast cancer recurrence to justify the omission of postoperative radiation therapy after lumpectomy. We excluded young patients from the trial, because young age has been considered a risk factor for local recurrence (de la Rochefordiere et al, 1993; Fowble et al, 1994), and selected for the study cancers of presumably low biological aggressiveness, as judged from a panel of commonly used prognostic factors. The great majority (93%) of all cancers were treated by only 4 surgeons, and we required at least 1 cm microscopically healthy tissue margin and preoperative mammography for study inclusion. In spite of these measures, lumpectomy without postoperative radiotherapy was associated with an increased cancer recurrence rate in the ipsilateral breast as compared with lumpectomy followed by postoperative radiotherapy. However, breast preservation, recurrence-free survival, and overall survival rates were similar and excellent in both study groups during a mean follow-up time of 6.7 years.

The 5-year cancer-specific survival rate in the present series was as high as 98%, suggesting that we were successful in selecting cancers of low biological aggressiveness. Despite this, the overall locoregional recurrence rate was 18.1% in the no-radiotherapy cohort, much higher than the 7.5% in the radiotherapy group. These figures do not appear to be different from those found in series where patients were selected for breast-conserving therapy primarily on the basis of tumour size (Fisher et al, 1995; Liljegren et al, 1999), suggesting that the biological factors which predict survival may not work equally well in the prediction of local recurrence.

There was no difference between the groups in the breast preservation rate. Recurrent or second ipsilateral breast cancers in patients randomly allocated to radiotherapy were usually treated with mastectomy, whereas the converse was the case in the no-radiotherapy group. The option for post-lumpectomy radiotherapy was still available for patients who had not received postoperative radiotherapy after lumpectomy, and treatment of recurrent or second cancer with limited surgery and irradiation balanced out the increased number of local breast cancer recurrences detected in the no-radiotherapy arm, resulting in a similar breast preservation rate in both treatment groups. Recurrent tumours may also be more difficult to diagnose early in the more dense and more fibrotic irradiated breast tissue than in the unirradiated breast, this possibly leading to larger tumour size at diagnosis and more often to mastectomy (Kearney and Morrow, 1995).

Although the mastectomy rates were similar in the two groups, the possible adverse prognostic influence of local recurrence and the resulting psychological distress should not be underestimated. In the present series with low-malignancy tumours the increased local relapse rate did not result in reduced cancer-specific or recurrence-free survival. On the other hand, distant metastases were more common in the radiotherapy group. The number of cases is, however, was too small to allow any firm conclusions. In a multivariate analysis from the National Surgical Adjuvant Breast and Bowel Project (NSABP) study B-06, which compared mastectomy, lumpectomy without irradiation, and lumpectomy with irradiation in the treatment of breast cancers 4 cm or less in diameter, the findings indicated that local recurrence is associated with a 3-fold increased relative risk of distant metastases, even when other prognostic factors are also considered (Fisher et al, 1991). Similar findings have been reported by others (Whelan et al, 1994).

Although the majority of patients receiving post-lumpectomy radiation therapy have good cosmetic outcome when treated by modern radiation therapy techniques (Dalberg et al, 1999), the therapy may contribute to breast fibrosis and cause nipple retraction. Thus omission of radiotherapy through careful patient selection might improve the cosmetic outcome. Omission of radiation therapy would significantly reduce the total cost of therapy. In the present series only 12.5% of the patients randomly assigned to the no-radiotherapy arm ever received radiotherapy, as compared to 100% of those who were randomized to the postoperative radiotherapy arm. However, no formal cost analysis was carried out,

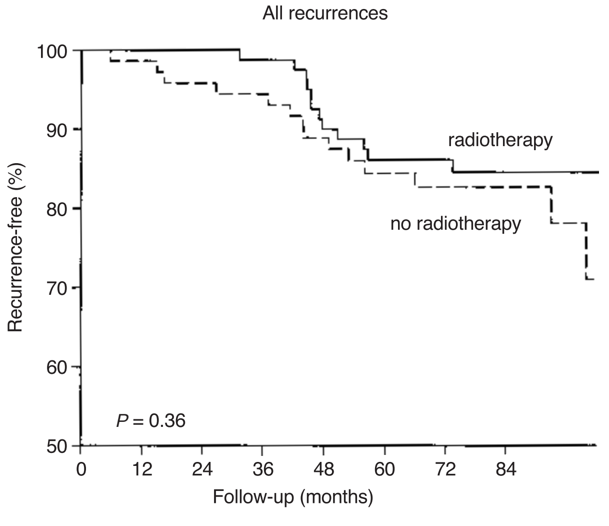

**Figure 3** Recurrence-free survival. Solid line, postoperative radiotherapy given; dashed line, no postoperative radiotherapy

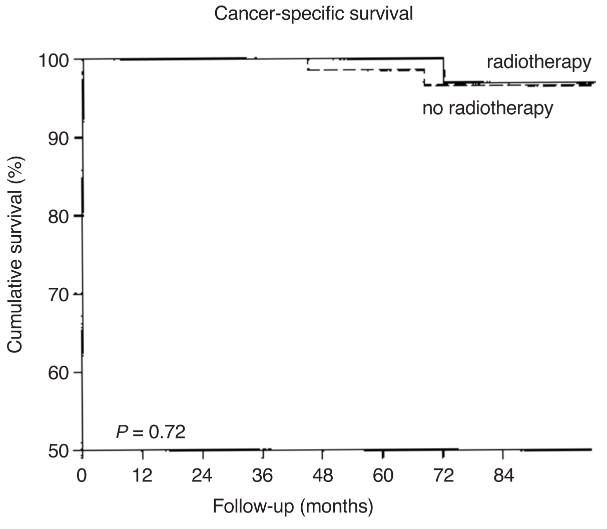

**Figure 4** Cancer-specific survival. Solid line, postoperative radiotherapy given; dashed line, no postoperative radiotherapy

© 2001 Cancer Research Campaign

**Table 3** Association of age at randomization and tumour size and histopathology with local recurrence

| Parameter | No local recurrence *n* (%) | Local recurrence *n* (%) | *P* value |
|---|---|---|---|
| Age | | | |
| <50 | 34 (83) | 7 (17) | |
| 50–59 | 58 (89) | 7 (11) | |
| ≥60 | 41 (89) | 5 (11) | 0.58 |
| Tumour size | | | |
| <10 mm | 54 (89) | 7 (11) | |
| ≥10 mm | 79 (87) | 12 (13) | 0.95 |
| Histologic type | | | |
| ductal | 101 (88) | 14 (12) | |
| lobular | 14 (88) | 2 (12) | 0.94 |
| special | 17 (85) | 3 (15) | |
| Histologic grade | | | |
| grade 1 | 90 (87) | 14 (13) | |
| grade 2 | 25 (86) | 4 (14) | 0.96[a] |
| not specified | 18 (95) | 1 (5) | |

[a](ductal vs. lobular).

and treatment of recurrences is associated with further costs in addition to those related to radiation therapy.

In summary, post-lumpectomy radiation therapy reduces the frequency of ipsilateral breast cancer recurrences even in women who have small (< 2 cm in diameter) node-negative breast cancer with several favourable prognostic features, and when the cancer has been removed with at least 1 cm margin. However, we obtained no evidence suggesting that postoperative radiotherapy improved the breast preservation rate or survival in this selected subgroup of patients. Commonly used prognostic factors may not be efficient in predicting the tendency of breast cancer to recur locally. Further studies using novel biological factors and new breast imaging techniques and aiming at identifying subgroups of breast cancer patients with a small risk of local recurrence after breast-preserving surgery are highly warranted.

### ACKNOWLEDGEMENTS

The authors are indebted to Mrs Kirsi Rouhento for help. This study was financially supported by the Finnish Cancer Society and the Finnish Breast Cancer Group.

### REFERENCES

Bloom HJG and Richardson WW (1957) Histological grading and prognosis in breast cancer. *Br J Cancer* **11**: 359–377

Clark RM, McCullogh PB, Levine MN, Lipa M, Wilkinson RH, Mahoney LJ, Basrur VR, Nair BD, McDermot RS, Wong CS and Corbett PJ1 (1992) Randomized clinical trial to assess the effectiveness of breast irriditation following lumpectomy and axillary dissection for node-negative breast cancer. *J Natl Cancer Inst* **84**: 683–689

Clarke DH and Martinez AA (1992) Identification of patients who are at high risk for locoregional breast cancer recurrence after conservative surgery and radiotherapy: A review article for surgeons, pathologists and radiation and medical oncologists. *J Clin Oncol* **10**: 474–483

Dalberg K, Eriksson E, Kanter L, Sandelin K, Liedberg A, Auer G, Thorstensson S, Fredriksson I, Johansson U and Rutqvist LE (1999) Biomarkers for local breast recurrence after breast-conservation – a nested case-control study. *Breast Cancer Res and Treat* **57**: 245–259

de la Rochefordiere A, Abner A, Silver B, Vicini F, Recht A and Harris JR (1992) Are cosmetic results following conservative surgery and radiation therapy for early breast cancer dependent on technique? *Int J radiat Oncol Biol Phys* **23**: 925–931

de la Rochefordiere A, Asselain B, Campana F, Scholl SM, Fenton J, Vilcoq JR, Durand JC, Pouillart P, Magdalenat H and Fourquet A (1993) Age as a prognostic factor in premenopausal breast carcinoma. *Lancet* **341**: 1039–1043

Early Breast Cancer Trialists Collaborative Group (1995) Effects of radiotherapy and surgery in early breast cancer. *N Engl J Med* **333**: 1444–1455

Fisher B, Anderson S, Fisher ER, Redmond C, Wickerham DL, Wolmark N, Mamounas EP, Deutsch M and Margolese R (1991) The significance of ipsilateral breast tumour recurrence after lumpectomy. *Lancet* **338**: 327–331

Fisher B, Andersson S, Redmond CK, Wolmark N, Wickerham DL and Cronin WM (1995) Reanalysis and results after 12 years of follow-up in a randomized clinical trial comparing total mastectomy with lumpectomy with or without irradiation in the treatment of breast cancer. *N Engl J Med* **333**: 1456–1461

Fisher B, Dignam J, Wolmark N, Mamounas E, Costantino J, Poller W, Fisher ER, Wickerham DL, Deutsch M, Margolese R, Dimitrov N and Kavanah M (1998) Lumpectomy and radiation therapy for the treatment of intraductal breast cancer: findings from National Surgical Adjuvant Breast and Bowel Project B-17. *J Clin Oncol* **16**: 441–452

Forrest AP, Stewart HJ, Everington D, Prescott RJ, McArdle CS, Harnett AN, Smith DC and George WD (1996) Randomized controlled trial of conservation therapy for breast cancer: 6-year analysis of the Scottish trial. *Lancet* **348**: 708–713

Fourquet A, Campana F, Zafrani B, Mosseri V, Vielh P, Durand JC and Vilcoq JR (1989) Prognostic factors of breast recurrence in conservative management of early breast cancer: A 25-year follow-up. *Int J Radiat Oncol Biol Phys* **17**: 719–725

Fowble BL, Schultz DJ, Overmoyer B, Solin LJ, Fox K, Jardines L, Orel S and Glick JH (1994) The influence of young age on outcome in early stage breast cancer. *Int J Radiat Oncol Biol Phys* **30**: 23–33

Gage I, Schnitt SJ, Nixon AJ, Silver B, Recht A, Troyan SL, Eberlein T, Love SM, Gelman R, Harris JR and Connolly JL (1996) Pathological margin involvement and the risk of recurrence in patients treated with breast conserving therapy. *Cancer* **78**: 1921–1928

Holland R, Connolly J, Gelman R, Mravunac M, Hendriks JH, Verbeek AL, Schnitt SJ, Silver B, Boyages J and Harris JR (1990) The presence of an extensive intraductal component (EIC) following a limited excision correlates with prominent residual disease in the remainder of the breast. *J Clin Oncol* **8**: 113–118

Julien J-P, Bijker N, Fentiman I, Peterse JL, Delledonne V, Rouanet P, Avril A, Sylvester R, Mignolet F, Bartelink H and Van Dongen JA (2000) Radiotherapy in breast-conserving treatment for ductal carcinoma in situ: first results of the EORTC randomised phase III trial 10853. *Lancet* **355**: 528–533

Kearney TJ and Morrow M (1995) Effect of re-excision on the success of breast-conserving surgery. *Ann Surg Oncol* **2**: 303–307

Kurtz J (1992) Factors influencing the risks of local recurrence in the breast. *Eur J Cancer* **28**: 660–666

Kurtz JM, Almaric R, Brandone H, Ayme Y, Jacquemier J, Pietra JC, Hans D, Pollet JF, Bressac C and Spitalier JM (1989). Local recurrence after breast-conserving surgery and radiotherapy. *Cancer* **63**: 1912–1917

Kurtz JM, Amalric R and Brandone H (1990) Risk factors for breast recurrence in premenopausal and postmenopausal patients with ductal cancers treated by conservation therapy. *Cancer* **65**: 1867–1878

Liljegren G, Holmberg L and Adami HO (1994) Sector resection with or without postoperative radiotherapy for stage I breast cancer: five-year results of a randomized trial. *J Natl Cancer Inst* **86**: 717–722

Liljegren G, Holmberg L, Bergh J, Lindgren A, Tabar L, Nordgren H and Adami HO (1999) 10-year results after sector resection with or without postoperative radiotherapy for stage I breast cancer: A randomized trial. *J Clin Oncol* **17**: 2326–2333

Locker AP, Ellis IO and Morgan DAL (1989) Factors influencing local recurrence after excision and radiotherapy for primary breast cancer. *Br J Surg* **76**: 890–894

Renton SC, Gazet JC, Ford HT, Corbishley C and Sutcliffe R (1996) The importance of resection margin in conservative surgery for breast cancer. *Eur J Surg Oncol* **22**: 17–22

Sinn HP, Anton HW, Magener A, von Fournier D, Bastert G and Otto HF (1998) Extensive and predominant in situ components in breast carcinoma: their influence on treatment results after breast-conserving therapy. *Eur J Cancer* **34**: 646–653

van Dongen JA, Bartelink H, Fentiman IS, Lerut T, Mignolet F, Olthuis G, van der Schueren E, Sylvester R, Tong D, Winter J and van Zijl K (1992) Factors influencing local relapse and survival and results of salvage treatment after breast-conserving therapy in operable breast cancer: EORTC trial 10801, breast

 *© 2001 Cancer Research Campaign*

conservation compared with mastectomy in TNM stage I and II breast cancer. *Eur J Cancer* **28A(4/5)**: 801–805
Veronesi U, Zucali R and Del Vecchio M (1985) Conservative treatment of breast cancer with the QU.A.RT. technique. *World J Surg* **9**: 676–681
Veronesi U, Luini A, Del Vecchio M, Greco M, Galimberti V, Merson M, Rilke F, Sacchini V, Saccozzi R, Savio T, Zucali R, Zurrida S and Sarvadori B (1993) Radiotherapy after breast-preserving surgery in women with localised cancer of the breast. *New Engl J Med* **328(22)**: 1587–1591
Veronesi U, Salvadori B, Luini A, Greco M, Saccozzi R, del Vecchio M, Mariani L, Zurrida S and Rilke F (1995) Breast conservation is a safe method in patients with small cancer of the breast: Long-term results of three randomized trials on 1,973 patients. *Eur J Cancer* **31A**: 1574–1579
Voogd AC, Peterse JL, Crommelin MA, Rutgers EJ, Botke G, Elkhuizen PH, van Geel AN, Hoekstra CJ, van Pel R, van de Vijver MJ and Coebergh JW (1999) Histological determinants for different types of local recurrence after breast-conserving therapy in invasive breast cancer. *Eur J Cancer* **35**: 1828–1837
Whelan T, Clark R, Roberts R, Levine M and Foster G (1994) Ipsilateral breast tumor recurrence post-lumpectomy is predictive of subsequent mortality: results from a randomized trial. *Int J Radiat Oncol Biol Phys* **30**: 11–16

*© 2001 Cancer Research Campaign*
